# New advances and trends in mass spectrometry instrumentation and technology, part II: introduction, data management and bioinformatics, collaboration and industry needs

**DOI:** 10.3389/fmolb.2026.1841641

**Published:** 2026-06-24

**Authors:** Andras Szeitz, Patrick Batoon, Paul R. S. Baker, LeRoy B. Martin

**Affiliations:** 1 Genome Science and Technology Program, University of British Columbia, Vancouver, BC, Canada; 2 Agilent Technologies, Inc., Santa Clara, CA, United States; 3 Sciex, Seattle, WA, United States; 4 Waters Corporation, Milford, MA, United States

**Keywords:** mass spectrometry, round-table discussion, Agilent Technologies, Inc., Sciex, Thermo Fisher Scientific, Waters Corporation

## 1 Introduction

Part I of this paper summarized the roundtable webinar discussion, which provided an Introduction and addressed questions on gaps in Single-Cell Analysis, Metabolomics, Lipidomics, and Multi-Omics Integration as experienced by major mass spectrometry vendors. In Part II, we extend this summary by examining Data Management and Bioinformatics, as well as Collaboration and Industry Needs from the perspectives of the mass spectrometry industry.

## 2 Data management and bioinformatics

### 2.1 What are the greatest challenges in mass spectrometry data standardization and reproducibility?

The mass spectrometry community have done a good job in defining what a mass spectrum is and establishing the core parameters that must be measured and reported. For example, once parameters, such as TIC ions, ion intensity, retention time, and CCS, etc., are obtained, we can express them in open formats like mzML with high fidelity. The harder part is not generating the data but ensuring that the world handles that data the same way. Vendors are accountable for the data their instruments generate, and everyone should have the same level of accountability for manipulating, interpreting, processing, and ultimately using that data. We have made progress in standardization of the data we utilize today. One such example is LIPID MAPS (Sud et al., 2007), which originated from using standard protocols on cell lines, culturing conditions, extraction procedures, instrument setup, expected results, nomenclature rules for reporting results, etc. That type of structured, shared framework exists across many other omics fields, and they are essential, because they encourage mass spectrometry vendors to incorporate best practices into their solutions and ensure those community expectations are met. Without aligning to those standards, our customers would not adopt those technologies. However, in small-molecule omics, challenges remain in normalization or standardization, mainly due to the lack of primary reference standards. As an example in lipidomics, we can tentatively identify something as a lipid, or perhaps a phosphocholine, or certain fatty acids, but we often operate somewhat blind. Without primary standards, we do not know the true ionization or fragmentation efficiency of a molecule, so we cannot derive exact concentrations. Instead, we rely on relative comparisons, i.e., sample A is twice the abundance of sample B, which is not ideal. Vendors are not typically the ones generating those standards, so progress may depend on collaboration with the scientific community to create these standards.

Mass spectrometric data formats are different for various instruments. Data acquired on one instrument may not be read on another instrument due to different file format. Proprietary metadata will probably always exist, but the community has been requesting common output options, and today, current instruments can generate a common data file format, i.e., mzML, which is shared, continually validated, and updated. In addition to the vendor-specific native formats, this is commonly available to the users. Vendors offer plug-ins, or they include it in their data acquisition options to export data into these universal file formats that can be loaded into third party data analysis software. Keeping the vendors informed and making that request is helpful and appreciated.

The two big non-profit software platforms, such as Skyline (MacLean et al., 2010) and MS Dial (Tsugawa et al., 2015) are good examples. They accept data from any vendor, particularly MS Dial which accommodates file formats, such as TOF OAD, TOF EAD, HPD, and this is how we may unify commercial vendors’ datasets. Enabling the community is a priority, so all vendors provide tools to load native raw formats directly into third-party software. One challenge with mzML is that converting data is never perfect, so assumptions get made, which may cause information to be lost and that is a concern. Making sure they can read the actual true native data format in the way that is intended is important.

## 3 Collaboration and industry needs

### 3.1 How can academia, industry, and regulators collaborate more efficiently to produce the highest quality of data without being over regulated?

I think the key to this is developing the academic industry relationships. Regulators come from both fields, and ultimately a strong industry-academia partnership will drive the regulation. Regulations often lag system or method development, so industry and academia partners can guide that process. The regulations do not have to come from the government. Certain organizations could set various standards and have various communities decide on best practices, method parameters, *etc.*, and how to analyze a data file in a specific way. A good example is the International Lipidomics Symposium, where a group of scientists created guidelines that journal editors should take into consideration when reviewing papers and listing core fundamentals that must be met before lipidomics data are taken seriously. This is good self-regulation, where academia and industry experts in the field define the requirements and vendors respond to the expectations in a working partnership without outside regulation, but mainly driven by the academic community, such as in International Lipidomics Symposium. It really goes back to how things develop. First, we need to recognize there is a problem, then develop an aligned solution. Everyone in the field shares the motivation to ensure mass spectrometry continues to solve real-world challenges. The technology can be applied across many different applications, and it is great fun to be involved in the process, making sure that these technologies can be leveraged appropriately in any use case, whether that is regulated or not.

A good example of academia-industry collaboration with regulatory oversight is the field of PFAS analysis. Academics were the first to discover the harmful health effects of PFAS compounds to the point where it became necessary to regulate them. The regulators issued guidance where the vendors need to be able to measure them at four parts per quadrillion level. Following that aspirational mandate, we had to live up to the challenge and have our mass spectrometers sensitive enough to be able to measure at that sensitivity level. This is one example for good harmonization, where we all got together and had to address an issue from different perspectives. In the end, we have to come up with a mass spectrometer that is sensitive enough to be able to do it on a routine basis. In some cases, regulators help drive the science by providing moonshot initiatives, setting really ambitious goals that pull technology forward instead of following behind. In regulated fields, the existence of clearly defined performance metrics is essential. The EPA guidelines in the United States (EPA, 2022; EPA, 2024) are a good example: they draw a firm line, and if an instrument cannot meet that threshold, it is not acceptable. From the vendor perspective, it is essential to have a goal that you have to reach in defining that performance requirement. In some cases, compounds behave differently, when certain analytes are easy to analyze and provide good results, yet others are more challenging yielding results with higher variance. However, laboratories need to remain compliant, and this may open new channels of communication with regulators for harmonization of regulatory guidelines. The recent changes are really a microcosm of what has happened in drug development in recent decades. Historically, pharmaceutical compounds were dominated by small molecules, which had relatively uniform analytical requirements. Over the last decade, however, biotherapeutics, such as antibodies, ADCs, mRNA, identified viruses, have advanced, incredibly, in complexity. Each analyte demands a different analytical requirement to analyze, validate, and vendors are under pressure to deliver instruments that function like a “Swiss army knife,” capable of handling these different permutations.

But building a commercial mass spectrometer is full of compromises, and the question becomes how we accommodate all these needs and still make a commercially viable product. This ties into the question of global regulatory alignment. Should the world operate under a single analytical standard rather than separate European, North American, or regional guidelines? It is not easy to comment, but once a standard becomes in use, others tend to follow. Vendors also naturally prioritize regulations from larger markets, such as North America or the USA/Canada, because instrument development is extremely expensive.

From a vendor perspective, we aim for the highest bar we expect to match, and there must be sufficient market incentive, such as financial or ethical, to make it worthwhile meeting those design requirements. Long development cycles add further uncertainty and considering the interesting interplay between what we need today and 5 years from now, we need to be cognizant when answering these crystal-ball questions. We should also be cautious about having a single global organization controlling analytical methods, because various regions and countries may have different motivations and values. Vendors will always strive for the highest standards, but standardization may be both a double-edged sword. Even in a well-established field like lipidomics, the community is surprisingly unstandardized on basic parameters, such as the type of column, chromatographic method, mass spectrometer, etc., to use and how to use it. The vendors should always have some room for creativity and flexibility, as a Swiss army knife to enable any user to obtain reliable data, but enough freedom for experts to optimize their methods fully. Historically, laboratories relied on a so-called “mass spec guru”, a person who could develop methods, verify compounds, and troubleshoot anything, etc. But developing a person with that level of expertise may take years, and success is not always guaranteed. This raises the question why vendors would not develop in-house experts who develop and validate methods that are shipped with each instrument? This would ensure the package will meet the regulatory compliance in most parts of the world. In fact, this already exists in certain sectors. Medical devices, for example, have to meet very strict regulatory requirements. Most vendors have medical devices that are validated for a particular task, and these workflows are very expensive to develop and usually apply to only one or two compounds. In certain cases, the users might say, “I do not want to measure vitamin D, instead, I want to measure this other compound”, and that is where the problems may start, but those instruments exist with all vendors.

That said, vendors have worked on solutions in non-regulated spaces. For example, Sciex introduced the lipidizer, which is a tailored solution for lipidomics, or Agilent may work on method workflow technologies on triple quadrupole systems that are built to streamline method development, Waters might be developing BioAccord, introducing tailored workflows in biopharma, and Thermo might be constructing specific workflows for various fields where a turnkey solution is required, instead of more expansive academic efforts of one instrument performing all tasks. And that is certainly an important part of the industry as well.
